# Supplementary figures and images for: An expanded toolkit for Drosophila gene tagging using synthesized homology donor constructs for CRISPR-mediated homologous recombination
Source: eLife. 2022 Jun 20;11:e76077. doi: 10.7554/eLife.76077 (PMC9239680; doi:10.7554/eLife.76077)

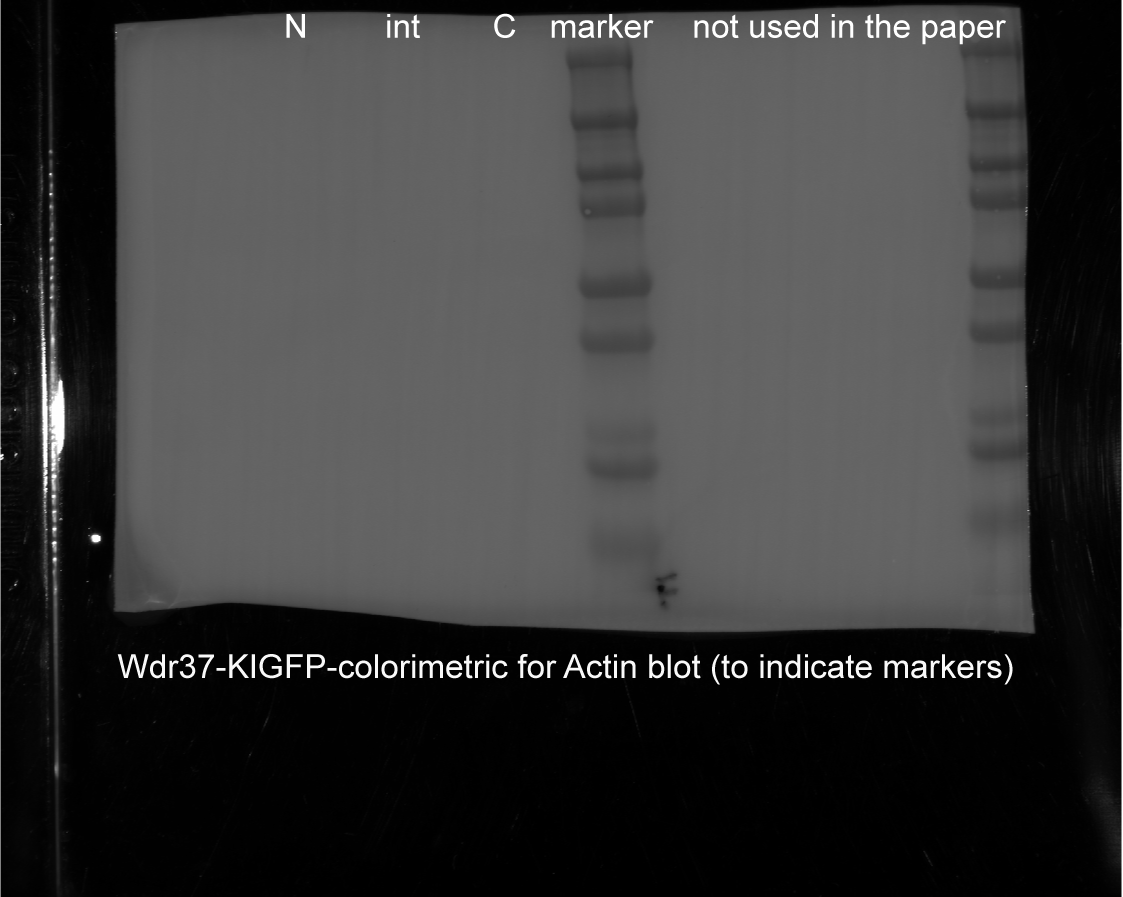

Supplement: Figure 5—source data 1. [file elife-76077-fig5-data1.zip › Figure5_source_data/Actin_colorimetric.tif]

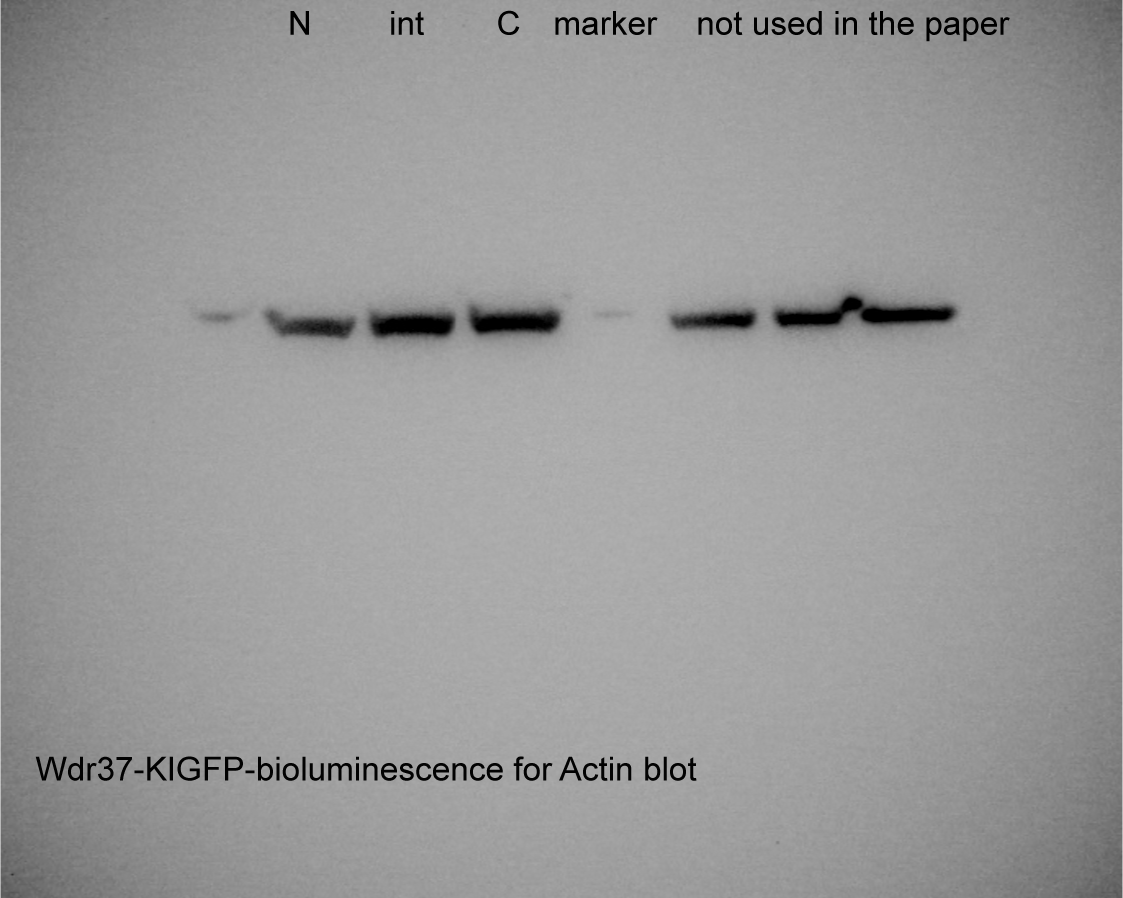

Supplement: Figure 5—source data 1. [file elife-76077-fig5-data1.zip › Figure5_source_data/Actin_bioluminescence.tif]

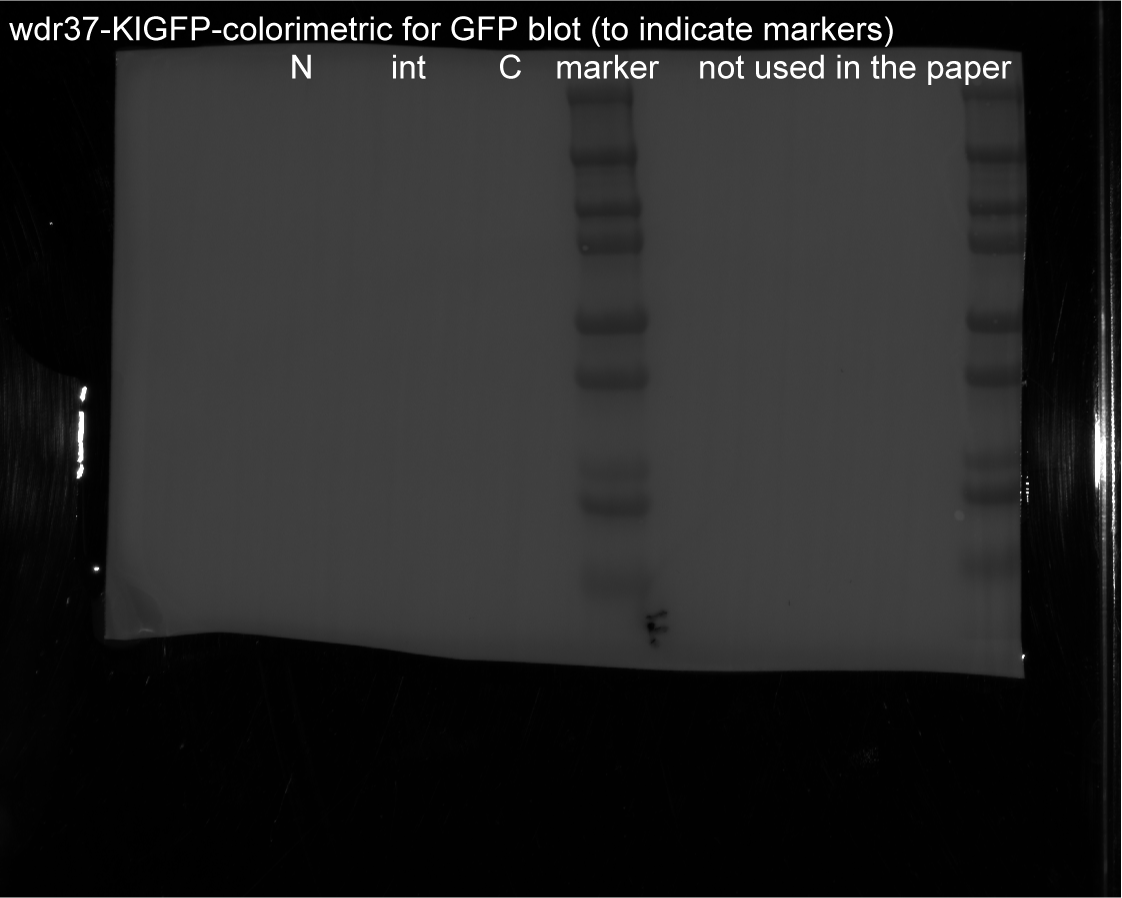

Supplement: Figure 5—source data 1. [file elife-76077-fig5-data1.zip › Figure5_source_data/GFP_colorimetric.tif]

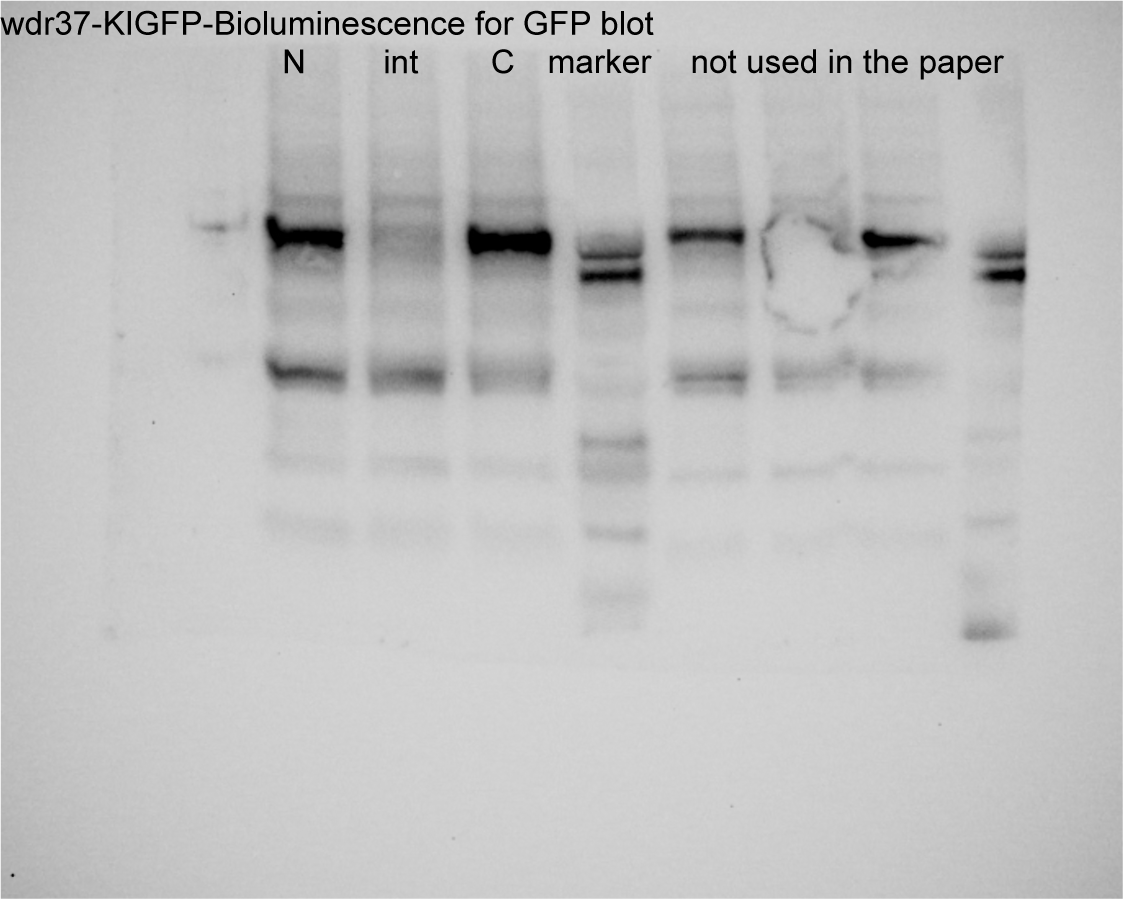

Supplement: Figure 5—source data 1. [file elife-76077-fig5-data1.zip › Figure5_source_data/GFP_bioluminescence.tif]
