# Supplementary material for: An expanded toolkit for Drosophila gene tagging using synthesized homology donor constructs for CRISPR-mediated homologous recombination
Source: eLife. 2022 Jun 20;11:e76077. doi: 10.7554/eLife.76077 (PMC9239680; doi:10.7554/eLife.76077)
Supplement: Supplementary file 3. — Step-by-step instructions and diagrams depicting construct design and cloning steps. Construct sequences and links to the digital construct sequence files are included in the protocol steps. [file elife-76077-supp3.docx]

**Supplementary methods Detailed cloning protocol and sequences**

**Design of new CRIMIC synthesis constructs:**

**Int200 strategy**

Design region:

**
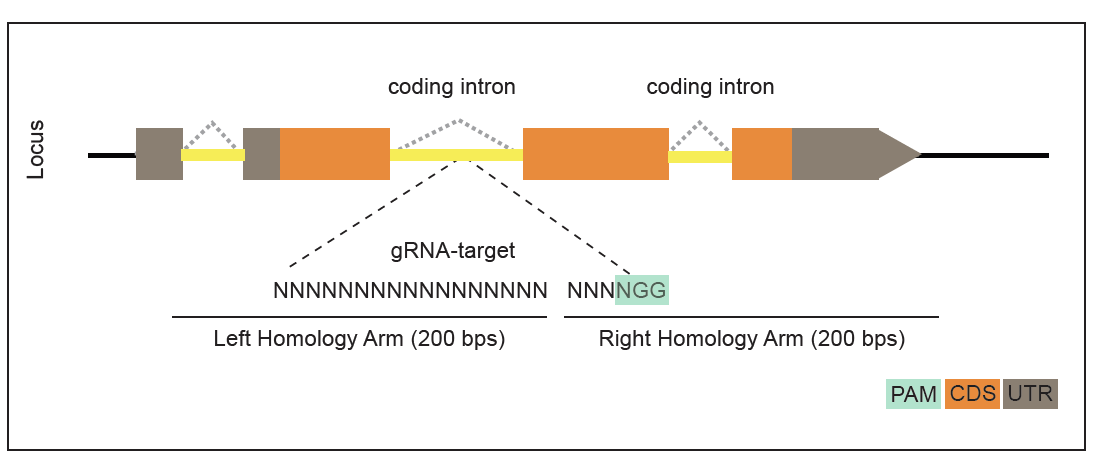
**

- 200 nucleotides on both sides of the cut site are copied in the scaffold file in the indicated regions (LHA for left homology arm, RHA for right homology arm) (int200_scaffold_BbsI file, in case the homology arms contain BbsI sites, BsaI-HF can be used for cloning. In that case int200_scaffold_BsaI scaffold file should be used for ordering the synthesis)


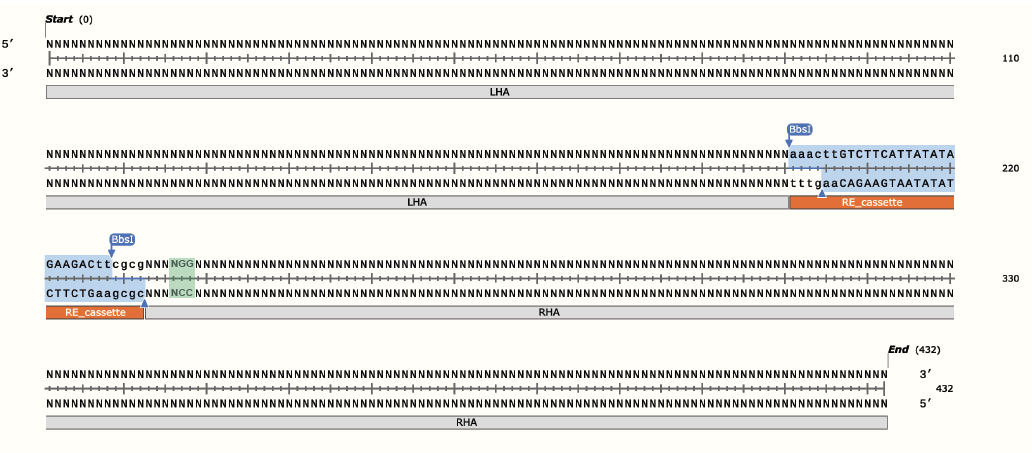


Scaffold sequence files can be found at:

int200_scaffold_bbsI:

<https://benchling.com/s/seq-Dhy16wJLeLnNSnP3c2O3?m=slm-Z1pBawgsXhAZuppvR6ZH>

int200_scaffold_bsaI:

https://benchling.com/s/seq-DYUJhqoCvyyGN2INjJ51?m=slm-8m3TiSGLRnxrfYor3dcc

Resulting DNA sequence is used for synthesis. The construct synthesis is ordered from Genewiz in “Value gene synthesis” format (at 4nM scale). While ordering, the following note is added in the ordering page “The constructs should be directionally cloned in pUC57_Kan_gw_OK vector in EcoRV site”. Genewiz uses Gibson assembly to integrate the synthesized fragments in the destination vector. Hence, the final orientation of the fragment can be specified. pUC57_Kan_gw_OK vector has the gRNA1 target sites on either side of EcoRV cut site and hence places the homology arms in between gRNA1 cut sites. In the backbone there is a U6:gRNA1 that linearizes the construct *in vivo*. There is no need to order the glycerol stocks. Ordering constructs in lyophilised format facilitates the downstream reaction set up.

The vector map before the synthesis and cloning of the homology arms:


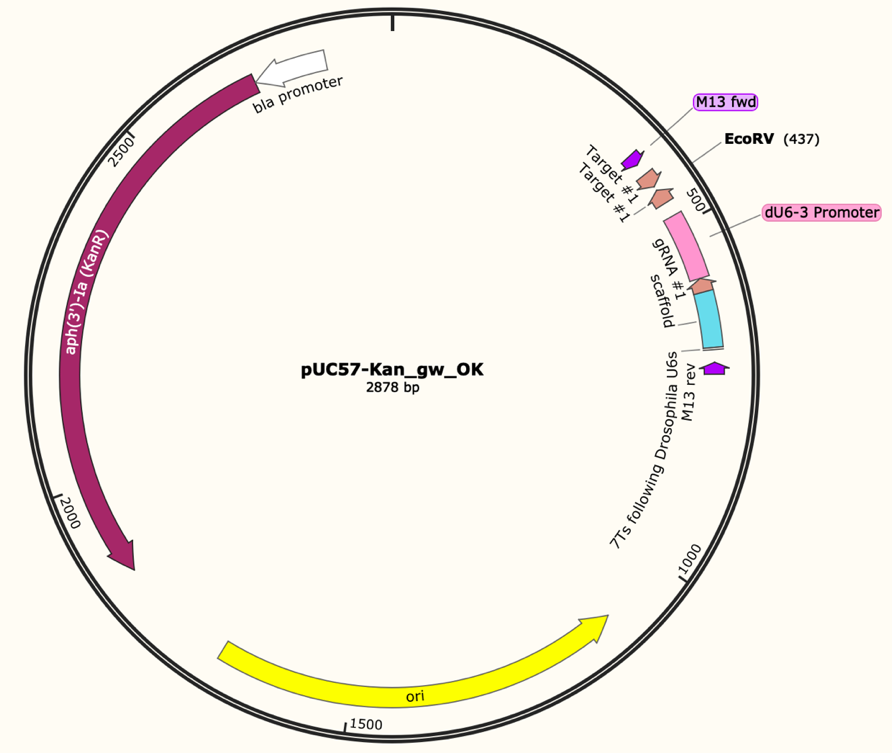


The vector map of pUC57-Kan_gw_OK after synthesis and cloning of homology arms (homology intermediate plasmid):


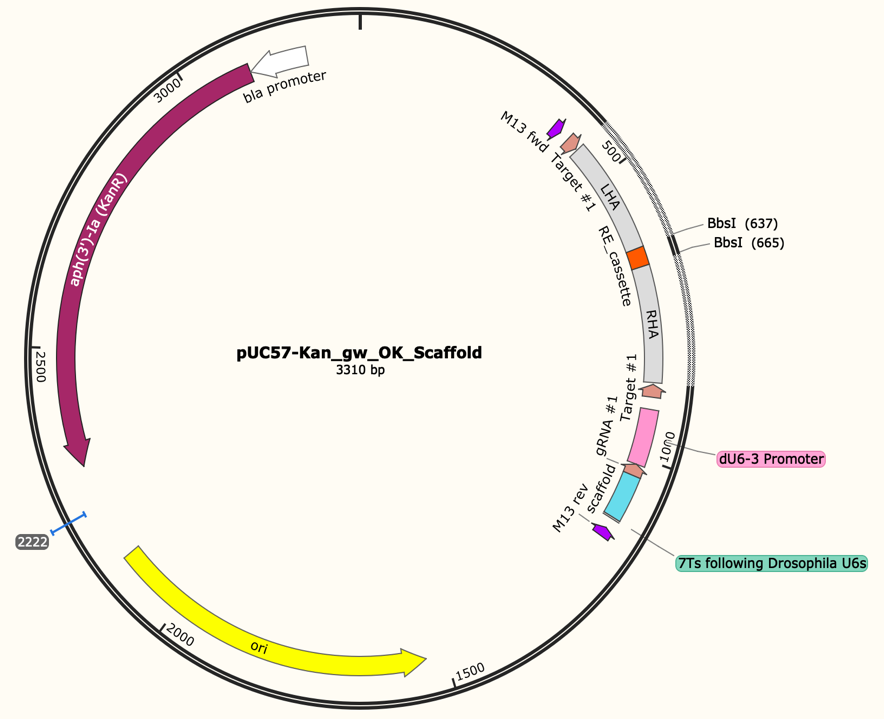


Vector sequence file for pUC57-Kan_gw_OK is available at: <https://benchling.com/s/seq-PBBFjaAttMgLQwcB8WQe?m=slm-EnGs2QGvq4izoCLxJ5UE>

**gRNA_int200 strategy**

Ordering gRNA_int200 constructs is similar to int200 construct with two exceptions:

-The target specific sgRNA sequences (20nts before the PAM sequence) should be added in the scaffold file in the indicated region.

-The synthesis should be conducted in pUC57_Kan_gw_OK2 vector in EcorV site directionally. pUC57_Kan_gw_OK2 vector contains the rest of the components of the homology donor construct.

The pUC57_Kan_gw_OK2 vector map before the synthesis and cloning of the homology arms:

**
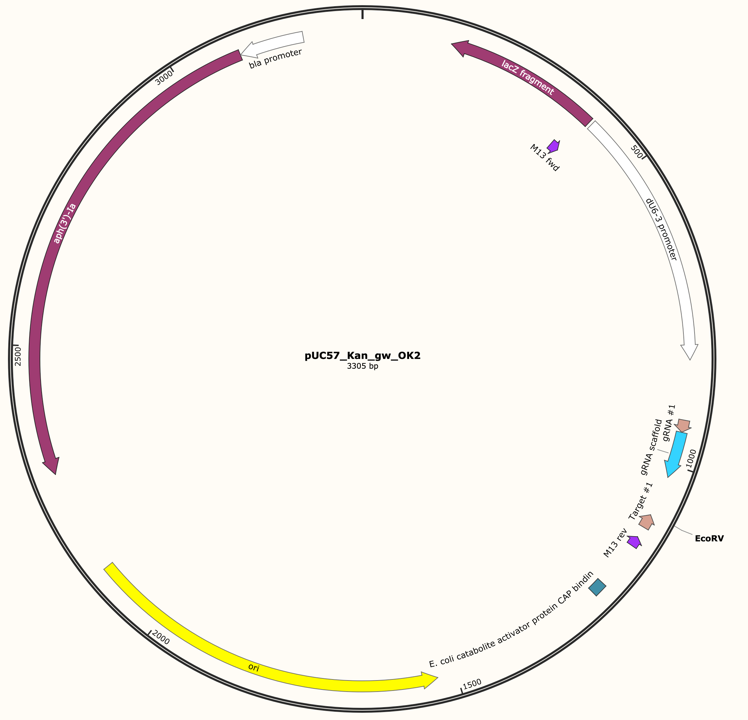
**

The vector map of pUC57-Kan_gw_OK2 after synthesis and cloning of homology arms (homology intermediate plasmid):


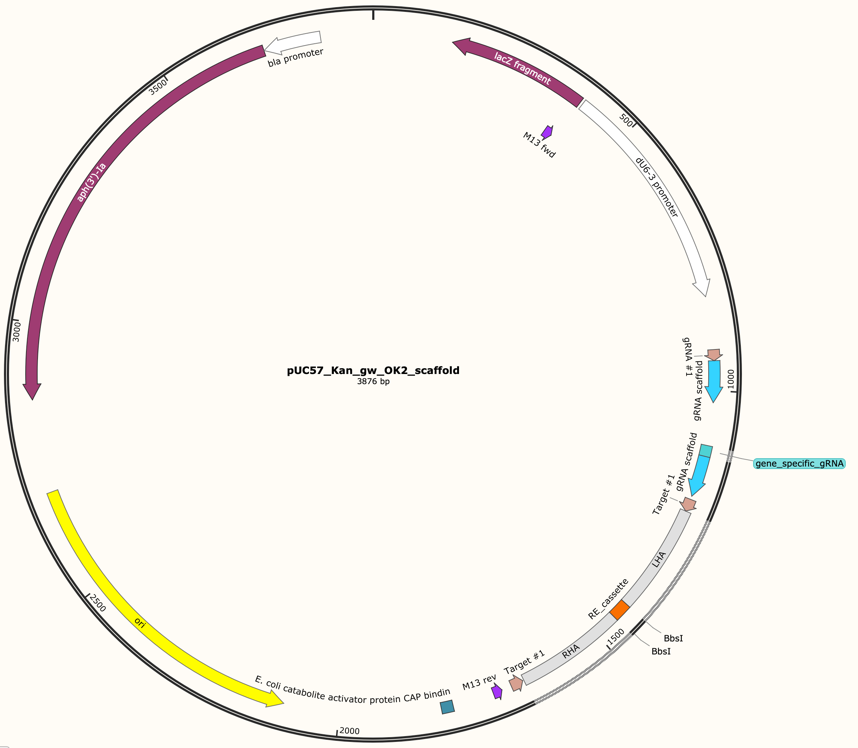


gRNA_int200_scaffold_bbsI:


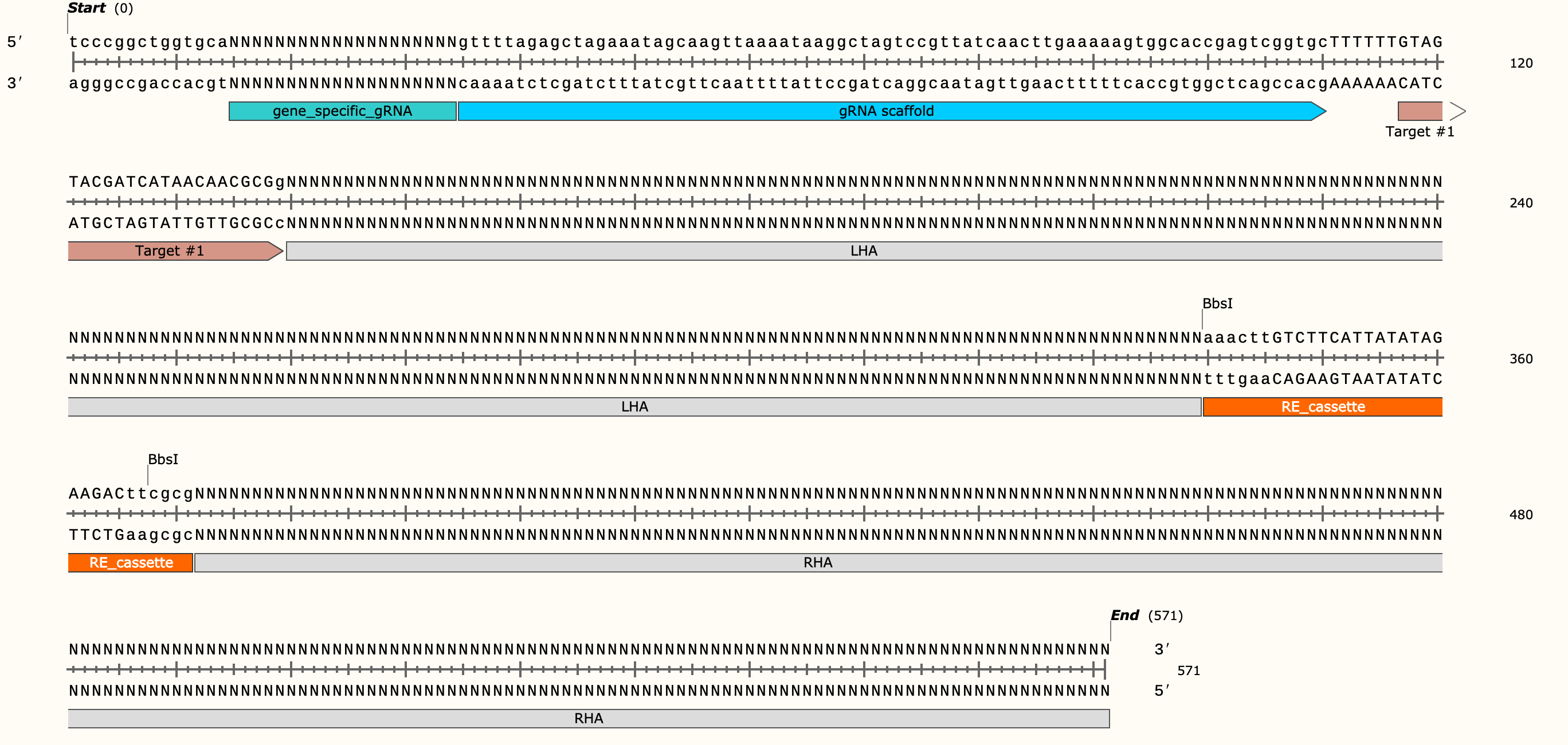


Part of pUC57_Kan_gw_OK2 vector without synthesized fragment:


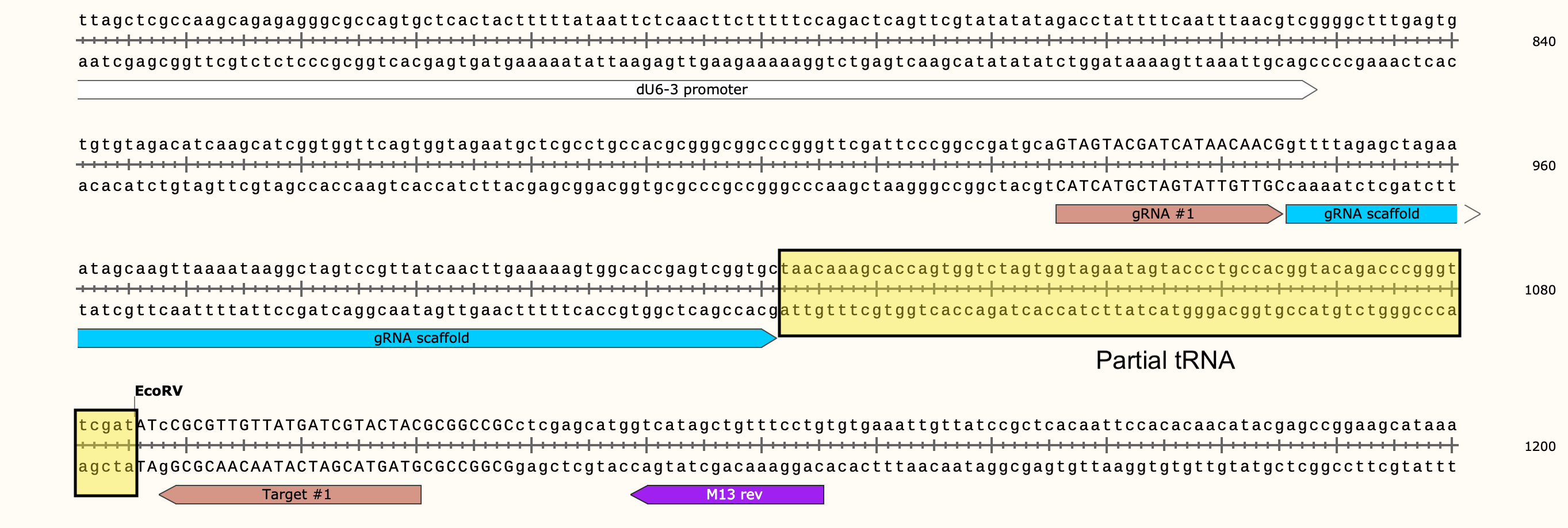


The same region pUC57_Kan_gw_OK2 vector with the synthesized fragment:


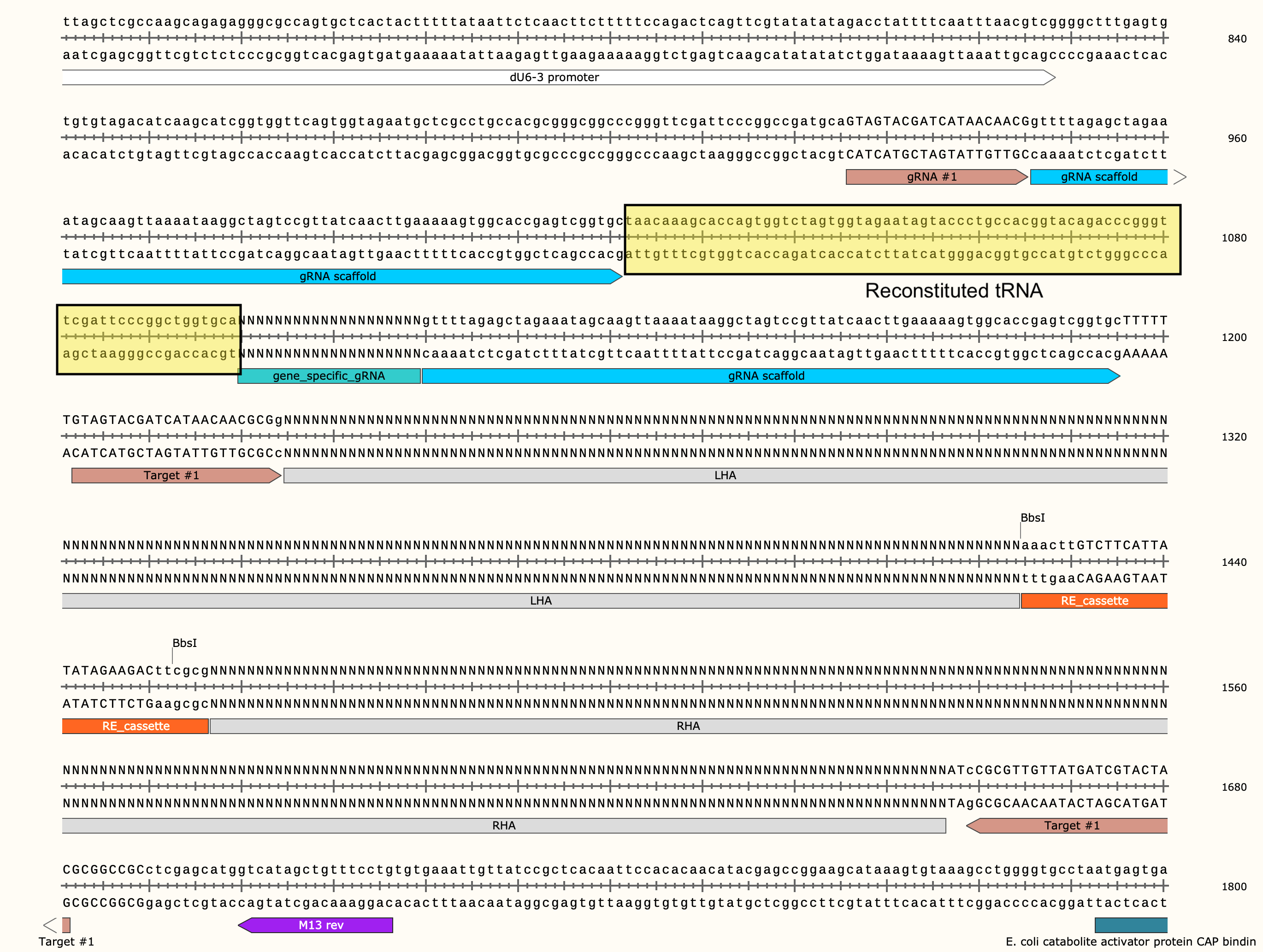


Reconstituted tRNA sequence corresponds to the sequence that separates multiple sgRNAs in pCFD5 vector (Port and Bullock, 2016).

pUC57_Kan_gw_OK2 sequence file is available at :

<https://benchling.com/s/seq-IkMBYajhoZbLulD1lRdi?m=slm-4oddMzCDRqyt4tEkcKoI>

Scaffold sequence files are available at:

gRNA_int200_scaffold_bbsI:

<https://benchling.com/s/seq-hPthMFIIR5BxgIc7CB9d?m=slm-XdfDHRd3d1tyZedvWwRS>

gRNA_int200_scaffold_bsaI:

<https://benchling.com/s/seq-wnAlYxz9HjlyuFEWqJyD?m=slm-2Ij2muR24ln1A8PoSQ8F>

For *KozakGAL4* constructs 2 sgRNAs are added in the synthesis reaction.


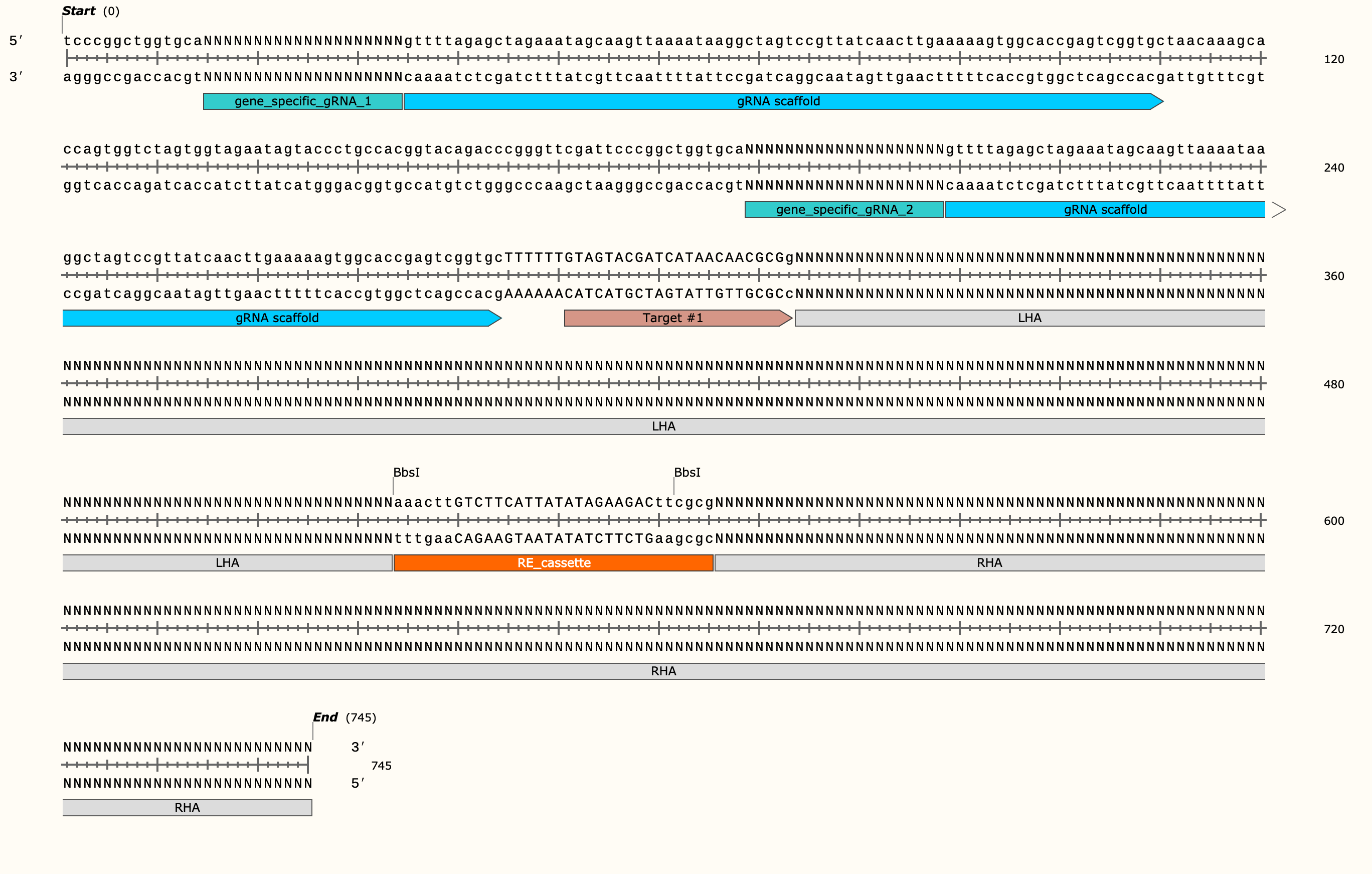


Scaffold sequence files are available at:

2XgRNA_int200_scaffold_bbsI:

<https://benchling.com/s/seq-WzBCh7QOA2MVX0xSbGtL?m=slm-c3uHmwefogsQqy61zmHC>

2XgRNA_int200_scaffold_bsaI:

<https://benchling.com/s/seq-GQN0KGiPGhyLaIgcXt1y?m=slm-vvY488sI2UzXqRslR8OJ>

**Cloning of constructs:**

- Genewiz synthesizes and clones the ordered fragments in pUC57_Kan_gw_OK or OK2 vectors and sends the resulting plasmids (homology intermediate plasmids) in lyophilized form. The lyophilized homology intermediate plasmids are resuspended in 53µl dH_2_O (Do not resuspend in TE buffer. Genewiz lyophilises the constructs in TE buffer. Resuspending in TE buffer decreases efficacy of downstream cloning applications).

-*T2AGAL4* or *KozakGAL4* (SIC) sequences are cloned in the homology intermediate plasmids to generate homology donor vectors. Proper SIC vector should be selected (correct phase of pM37 for *T2AGAL4* or pM37_KozakGAL4 or pM37_SA_KozakGAL4)

The reaction is set up as follows (a master mix can be prepared for cloning multiple homology donors with the same SIC):

- 1µl pM37-phase X* (290 ng/µl) or 1 µl pM37_KozakGAL4 (265 ng/µl)
- 2.5 µl 10X T4 DNA ligase buffer (NEB B0202S)
- 0.5 µl T4 DNA ligase (NEB M0202L)
- 1 µl Restriction enzyme (BbsI_HF (NEB R3733L) or BsaI_HFv2 (NEB R3559L)
- 19 µl of dH_2_O

The master mix is distributed in PCR tubes ( 24µl/sample) and 1 µl of reconstituted homology intermediate plasmid is added to the master mix.

* Phase selection is done by following the reading frame of the gene at the end of the preceding exon. If the last codon in the preceeding exon is complete (NNN-intron-NNN), the phase is p0. If one of the nucleotide of the codon is in the preceeding exon and two are in the following exon (N-intron-NN) the phase is p1. If two nucleotides of the codon is in the preceeding exon and one in the following exon (NN-intron-N) the phase is p2. It is crucial to select the correct phase to avoid a frameshift mutation.

-The reactions are incubated in a Thermocycler:

- 37°C 5 minutes
- 16°C 5 minutes
- Go to 1 30 times
- 65°C 20 minutes
- 8°C hold

The reactions can be left in the thermocycler overnight.

- An additional digestion step is done to remove self ligating homology intermediare plasmid backbones by adding:

- 19.5 µl dH_2_O
- 5 µl 10X Cutsmart buffer
- 0.5 µl BbsI or BsaI_HFv2 (the enzyme used for the cloning reaction)

The restriction mix can also be prepared as a mastermix and distributed to each sample 25µl/sample. The final digestion reactions are incubated for 30 minutes in 37°C incubator or thermocycler.

-After final digestion, the cloning products are transformed in 50 µl chemocompetent DH5-alpha. Selection antibiotic of homology donor plasmids is Kanamycin, hence 1-hour recovery is necessary after heatshock. Transformed bacteria are plated on LB plates with Kanamycin. Bacteria are incubated at 37°C overnight.

-(Optional) Next day, colony PCR can be conducted to preselect colonies that contain the correct homology donor plasmids using M13F_Long_for CRIMIC_ch_rev primers:

M13F_long_for gacgttgtaaaacgacggccag

CRIMIC_ch_rev gcggaagagagataaatcggttg

A colony is picked from the cloning plate using an autoclaved pipette tip, transferred on a gridded plate to copy the colony by touching the pipette tip to the plate and added to PCR mix by dipping the same pipette tip.

PCR conditions

0.2ul M13F_long_for

0.2ul CRIMIC_ch_rev

12.1 ul dH_2_O

12.5 ul OneTaq Quick-Load 2X Master Mix (M0486L)

Rxn: 94°C 30 sec

94°C 30 sec |

58°C 30 sec | 34 cycles

68°C 30 sec |

68°C 5 minutes

8°C Hold

-The positive colonies will show ~630 bps amplicon.

- The positive colonies are incubated in 5 ml of LB+Kanamycin and miiniprepped using Qiaprep Spin Miniprep kit (Qiagen 27106).

-Resulting DNA is sequenced using M13Reverse and intseq_forward primers (GTTCGATTCCCGGCCGATG). This step is highly recommended since we have noticed the EGFP coding sequences to be missing in occasional cloning products. Picking a different colony usually fixes the problem.

Vector sequences:

pUC57_Kan_gw_OK

TCGCGCGTTTCGGTGATGACGGTGAAAACCTCTGACACATGCAGCTCCCGGAGACGGTCACAGCTTGTCTGTAAGCGGATGCCGGGAGCAGACAAGCCCGTCAGGGCGCGTCAGCGGGTGTTGGCGGGTGTCGGGGCTGGCTTAACTATGCGGCATCAGAGCAGATTGTACTGAGAGTGCACCATATGCGGTGTGAAATACCGCACAGATGCGTAAGGAGAAAATACCGCATCAGGCGCCATTCGCCATTCAGGCTGCGCAACTGTTGGGAAGGGCGATCGGTGCGGGCCTCTTCGCTATTACGCCAGCTGGCGAAAGGGGGATGTGCTGCAAGGCGATTAAGTTGGGTAACGCCAGGGTTTTCCCAGTCACGACGTTGTAAAACGACGGCCAGTGAATTGACGCGTATTGGGTAGTACGATCATAACAACGCGGATATCCGCGTTGTTATGATCGTACTACCCAATGGCGCGCCGAGCTTGGAAGCAGAGAGGGCGCCAGTGCTCACTACTTTTTATAATTCTCAACTTCTTTTTCCAGACTCAGTTCGTATATATAGACCTATTTTCAATTTAACGTCGTAGTACGATCATAACAACGGTTTTAGAGCTAGAAATAGCAAGTTAAAATAAGGCTAGTCCGTTATCAACTTGAAAAAGTGGCACCGAGTCGGTGCTTTTTTTGCGGCCGCCTCGAGCATGGTCATAGCTGTTTCCTGTGTGAAATTGTTATCCGCTCACAATTCCACACAACATACGAGCCGGAAGCATAAAGTGTAAAGCCTGGGGTGCCTAATGAGTGAGCTAACTCACATTAATTGCGTTGCGCTCACTGCCCGCTTTCCAGTCGGGAAACCTGTCGTGCCAGCTGCATTAATGAATCGGCCAACGCGCGGGGAGAGGCGGTTTGCGTATTGGGCGCTCTTCCGCTTCCTCGCTCACTGACTCGCTGCGCTCGGTCGTTCGGCTGCGGCGAGCGGTATCAGCTCACTCAAAGGCGGTAATACGGTTATCCACAGAATCAGGGGATAACGCAGGAAAGAACATGTGAGCAAAAGGCCAGCAAAAGGCCAGGAACCGTAAAAAGGCCGCGTTGCTGGCGTTTTTCCATAGGCTCCGCCCCCCTGACGAGCATCACAAAAATCGACGCTCAAGTCAGAGGTGGCGAAACCCGACAGGACTATAAAGATACCAGGCGTTTCCCCCTGGAAGCTCCCTCGTGCGCTCTCCTGTTCCGACCCTGCCGCTTACCGGATACCTGTCCGCCTTTCTCCCTTCGGGAAGCGTGGCGCTTTCTCATAGCTCACGCTGTAGGTATCTCAGTTCGGTGTAGGTCGTTCGCTCCAAGCTGGGCTGTGTGCACGAACCCCCCGTTCAGCCCGACCGCTGCGCCTTATCCGGTAACTATCGTCTTGAGTCCAACCCGGTAAGACACGACTTATCGCCACTGGCAGCAGCCACTGGTAACAGGATTAGCAGAGCGAGGTATGTAGGCGGTGCTACAGAGTTCTTGAAGTGGTGGCCTAACTACGGCTACACTAGAAGAACAGTATTTGGTATCTGCGCTCTGCTGAAGCCAGTTACCTTCGGAAAAAGAGTTGGTAGCTCTTGATCCGGCAAACAAACCACCGCTGGTAGCGGTGGTTTTTTTGTTTGCAAGCAGCAGATTACGCGCAGAAAAAAAGGATCTCAAGAAGATCCTTTGATCTTTTCTACGGGGTCTGACGCTCAGTGGAACGAAAACTCACGTTAAGGGATTTTGGTCATGAGATTATCAAAAAGGATCTTCACCTAGATCCTTTTAAATTAAAAATGAAGTTTTAAATCAATCTAAAGTATATATGAGTAAACTTGGTCTGACAGTTAGAAAAACTCATCGAGCATCAAATGAAACTGCAATTTATTCATATCAGGATTATCAATACCATATTTTTGAAAAAGCCGTTTCTGTAATGAAGGAGAAAACTCACCGAGGCAGTTCCATAGGATGGCAAGATCCTGGTATCGGTCTGCGATTCCGACTCGTCCAACATCAATACAACCTATTAATTTCCCCTCGTCAAAAATAAGGTTATCAAGTGAGAAATCACCATGAGTGACGACTGAATCCGGTGAGAATGGCAAAAGTTTATGCATTTCTTTCCAGACTTGTTCAACAGGCCAGCCATTACGCTCGTCATCAAAATCACTCGCATCAACCAAACCGTTATTCATTCGTGATTGCGCCTGAGCGAGACGAAATACGCGATCGCTGTTAAAAGGACAATTACAAACAGGAATCGAATGCAACCGGCGCAGGAACACTGCCAGCGCATCAACAATATTTTCACCTGAATCAGGATATTCTTCTAATACCTGGAATGCTGTTTTCCCAGGGATCGCAGTGGTGAGTAACCATGCATCATCAGGAGTACGGATAAAATGCTTGATGGTCGGAAGAGGCATAAATTCCGTCAGCCAGTTTAGTCTGACCATCTCATCTGTAACATCATTGGCAACGCTACCTTTGCCATGTTTCAGAAACAACTCTGGCGCATCGGGCTTCCCATACAATCGATAGATTGTCGCACCTGATTGCCCGACATTATCGCGAGCCCATTTATACCCATATAAATCAGCATCCATGTTGGAATTTAATCGCGGCCTAGAGCAAGACGTTTCCCGTTGAATATGGCTCATACTCTTCCTTTTTCAATATTATTGAAGCATTTATCAGGGTTATTGTCTCATGAGCGGATACATATTTGAATGTATTTAGAAAAATAAACAAATAGGGGTTCCGCGCACATTTCCCCGAAAAGTGCCACCTGACGTCTAAGAAACCATTATTATCATGACATTAACCTATAAAAATAGGCGTATCACGAGGCCCTTTCGTC

sgRNA1_target sgRNA1 M13Frw primer U6-3 promoter sgRNA scaffold

M13rev primer Ori KanR bla promoter underlined sequences are in -strand

pUC57_Kan_gw_OK2

TCGCGCGTTTCGGTGATGACGGTGAAAACCTCTGACACATGCAGCTCCCGGAGACGGTCACAGCTTGTCTGTAAGCGGATGCCGGGAGCAGACAAGCCCGTCAGGGCGCGTCAGCGGGTGTTGGCGGGTGTCGGGGCTGGCTTAACTATGCGGCATCAGAGCAGATTGTACTGAGAGTGCACCATATGCGGTGTGAAATACCGCACAGATGCGTAAGGAGAAAATACCGCATCAGGCGCCATTCGCCATTCAGGCTGCGCAACTGTTGGGAAGGGCGATCGGTGCGGGCCTCTTCGCTATTACGCCAGCTGGCGAAAGGGGGATGTGCTGCAAGGCGATTAAGTTGGGTAACGCCAGGGTTTTCCCAGTCACGACGTTGTAAAACGACGGCCAGTGAATTGACGCGTTTTTTTGCTCACCTGTGATTGCTCCTACTCAAATACAAAAACATCAAATTTTCTGTCAATAAAGCATATTTATTTATATTTATTTTACAGGAAAGAATTCCTTTTAAAGTGTATTTTAACCTATAATGAAAAACGATTAAAAAAAATACATAAAATAATTCGAAAATTTTTGAATAGCCCAGGTTGATAAAAATTCATTTCATACGTTTTATAACTTATGCCCCTAAGTATTTTTTGACCATAGTGTTTCAATTCTACATTAATTTTACAGAGTAGAATGAAACGCCACCTACTCAGCCAAGAGGCGAAAAGGTTAGCTCGCCAAGCAGAGAGGGCGCCAGTGCTCACTACTTTTTATAATTCTCAACTTCTTTTTCCAGACTCAGTTCGTATATATAGACCTATTTTCAATTTAACGTCGGGGCTTTGAGTGTGTGTAGACATCAAGCATCGGTGGTTCAGTGGTAGAATGCTCGCCTGCCACGCGGGCGGCCCGGGTTCGATTCCCGGCCGATGCAGTAGTACGATCATAACAACGGTTTTAGAGCTAGAAATAGCAAGTTAAAATAAGGCTAGTCCGTTATCAACTTGAAAAAGTGGCACCGAGTCGGTGCTAACAAAGCACCAGTGGTCTAGTGGTAGAATAGTACCCTGCCACGGTACAGACCCGGGTTCGATATCCGCGTTGTTATGATCGTACTACGCGGCCGCCTCGAGCATGGTCATAGCTGTTTCCTGTGTGAAATTGTTATCCGCTCACAATTCCACACAACATACGAGCCGGAAGCATAAAGTGTAAAGCCTGGGGTGCCTAATGAGTGAGCTAACTCACATTAATTGCGTTGCGCTCACTGCCCGCTTTCCAGTCGGGAAACCTGTCGTGCCAGCTGCATTAATGAATCGGCCAACGCGCGGGGAGAGGCGGTTTGCGTATTGGGCGCTCTTCCGCTTCCTCGCTCACTGACTCGCTGCGCTCGGTCGTTCGGCTGCGGCGAGCGGTATCAGCTCACTCAAAGGCGGTAATACGGTTATCCACAGAATCAGGGGATAACGCAGGAAAGAACATGTGAGCAAAAGGCCAGCAAAAGGCCAGGAACCGTAAAAAGGCCGCGTTGCTGGCGTTTTTCCATAGGCTCCGCCCCCCTGACGAGCATCACAAAAATCGACGCTCAAGTCAGAGGTGGCGAAACCCGACAGGACTATAAAGATACCAGGCGTTTCCCCCTGGAAGCTCCCTCGTGCGCTCTCCTGTTCCGACCCTGCCGCTTACCGGATACCTGTCCGCCTTTCTCCCTTCGGGAAGCGTGGCGCTTTCTCATAGCTCACGCTGTAGGTATCTCAGTTCGGTGTAGGTCGTTCGCTCCAAGCTGGGCTGTGTGCACGAACCCCCCGTTCAGCCCGACCGCTGCGCCTTATCCGGTAACTATCGTCTTGAGTCCAACCCGGTAAGACACGACTTATCGCCACTGGCAGCAGCCACTGGTAACAGGATTAGCAGAGCGAGGTATGTAGGCGGTGCTACAGAGTTCTTGAAGTGGTGGCCTAACTACGGCTACACTAGAAGAACAGTATTTGGTATCTGCGCTCTGCTGAAGCCAGTTACCTTCGGAAAAAGAGTTGGTAGCTCTTGATCCGGCAAACAAACCACCGCTGGTAGCGGTGGTTTTTTTGTTTGCAAGCAGCAGATTACGCGCAGAAAAAAAGGATCTCAAGAAGATCCTTTGATCTTTTCTACGGGGTCTGACGCTCAGTGGAACGAAAACTCACGTTAAGGGATTTTGGTCATGAGATTATCAAAAAGGATCTTCACCTAGATCCTTTTAAATTAAAAATGAAGTTTTAAATCAATCTAAAGTATATATGAGTAAACTTGGTCTGACAGTTAGAAAAACTCATCGAGCATCAAATGAAACTGCAATTTATTCATATCAGGATTATCAATACCATATTTTTGAAAAAGCCGTTTCTGTAATGAAGGAGAAAACTCACCGAGGCAGTTCCATAGGATGGCAAGATCCTGGTATCGGTCTGCGATTCCGACTCGTCCAACATCAATACAACCTATTAATTTCCCCTCGTCAAAAATAAGGTTATCAAGTGAGAAATCACCATGAGTGACGACTGAATCCGGTGAGAATGGCAAAAGTTTATGCATTTCTTTCCAGACTTGTTCAACAGGCCAGCCATTACGCTCGTCATCAAAATCACTCGCATCAACCAAACCGTTATTCATTCGTGATTGCGCCTGAGCGAGACGAAATACGCGATCGCTGTTAAAAGGACAATTACAAACAGGAATCGAATGCAACCGGCGCAGGAACACTGCCAGCGCATCAACAATATTTTCACCTGAATCAGGATATTCTTCTAATACCTGGAATGCTGTTTTCCCAGGGATCGCAGTGGTGAGTAACCATGCATCATCAGGAGTACGGATAAAATGCTTGATGGTCGGAAGAGGCATAAATTCCGTCAGCCAGTTTAGTCTGACCATCTCATCTGTAACATCATTGGCAACGCTACCTTTGCCATGTTTCAGAAACAACTCTGGCGCATCGGGCTTCCCATACAATCGATAGATTGTCGCACCTGATTGCCCGACATTATCGCGAGCCCATTTATACCCATATAAATCAGCATCCATGTTGGAATTTAATCGCGGCCTAGAGCAAGACGTTTCCCGTTGAATATGGCTCATACTCTTCCTTTTTCAATATTATTGAAGCATTTATCAGGGTTATTGTCTCATGAGCGGATACATATTTGAATGTATTTAGAAAAATAAACAAATAGGGGTTCCGCGCACATTTCCCCGAAAAGTGCCACCTGACGTCTAAGAAACCATTATTATCATGACATTAACCTATAAAAATAGGCGTATCACGAGGCCCTTTCGTC

Intseq_forward sgRNA1_target sgRNA1 part of tRNA sequence M13Frw primer

U6-3promoter sgRNA scaffold M13rev primer Ori KanR bla promoter underlined sequences are in -strand

pM37_KozakGAL4

CTAAATTGTAAGCGTTAATATTTTGTTAAAATTCGCGTTAAATTTTTGTTAAATCAGCTCATTTTTTAACCAATAGGCCGAAATCGGCAAAATCCCTTATAAATCAAAAGAATAGACCGAGATAGGGTTGAGTGTTGTTCCAGTTTGGAACAAGAGTCCACTATTAAAGAACGTGGACTCCAACGTCAAAGGGCGAAAAACCGTCTATCAGGGCGATGGCCCACTACGTGAACCATCACCCTAATCAAGTTTTTTGGGGTCGAGGTGCCGTAAAGCACTAAATCGGAACCCTAAAGGGAGCCCCCGATTTAGAGCTTGACGGGGAAAGCCGGCGAACGTGGCGAGAAAGGAAGGGAAGAAAGCGAAAGGAGCGGGCGCTAGGGCGCTGGCAAGTGTAGCGGTCACGCTGCGCGTAACCACCACACCCGCCGCGCTTAATGCGCCGCTACAGGGCGCGTCCCATTCGCCATTCAGGCTGCGCAACTGTTGGGAAGGGCGATCGGTGCGGGCCTCTTCGCTATTACGCCAGCTGGCGAAAGGGGGATGTGCTGCAAGGCGATTAAGTTGGGTAACGCCAGGGTTTTCCCAGTCACGACGTTGTAAAACGACGGCCAGTGAGCGCGCGTAATACGACTCACTATAGGGCGAATTGGAGCTCCACCGCGGTGGCGTCTCTAAACGGTCTCTAAACGAAGACTTAAACCAAAATGAAGCTGCTGAGCAGCATCGAGCAGGCCTGCGATATCTGCCGCCTGAAGAAGCTGAAGTGCAGCAAGGAGAAGCCCAAGTGCGCCAAGTGCCTGAAGAACAACTGGGAGTGCCGCTACAGCCCCAAGACCAAGCGCAGCCCCCTGACCCGCGCCCACCTGACCGAGGTGGAGAGCCGCCTGGAGCGCCTGGAGCAGCTGTTCCTGCTGATCTTCCCCCGCGAGGATCTGGATATGATCCTGAAGATGGATAGCCTGCAGGATATCAAGGCCCTGCTGACCGGCCTGTTCGTGCAGGATAACGTGAACAAGGATGCCGTGACCGATCGCCTGGCCAGCGTGGAAACCGATATGCCCCTGACCCTGCGCCAGCACCGCATCAGCGCCACCAGCAGCAGCGAGGAGAGCAGCAACAAGGGCCAGCGCCAGCTGACCGTGAGCATCGATAGCGCCGCCCACCACGATAACAGCACCATCCCCCTGGATTTCATGCCCCGCGATGCCCTGCACGGCTTCGATTGGAGCGAGGAGGATGATATGAGCGATGGCCTGCCCTTCCTGAAAACCGATCCCAACAACAACGGCTTCTTCGGCGATGGCAGCCTGCTGTGCATCCTGCGCAGCATCGGCTTCAAGCCCGAGAACTACACCAACAGCAACGTGAACCGCCTGCCCACCATGATCACCGATCGCTACACCCTGGCCAGCCGCAGCACCACCAGCCGCCTGCTGCAGAGCTACCTGAACAACTTCCACCCCTACTGCCCCATCGTGCACAGCCCCACCCTGATGATGCTGTACAACAACCAGATCGAGATCGCCAGCAAGGATCAGTGGCAGATCCTGTTCAACTGCATCCTGGCCATCGGCGCCTGGTGCATCGAGGGCGAGAGCACCGATATCGATGTGTTCTACTACCAGAACGCCAAGAGCCACCTGACCAGCAAGGTGTTCGAGAGCGGCAGCATCATCCTGGTGACCGCCCTGCACCTGCTGAGCCGCTACACCCAGTGGCGCCAAAAGACCAACACCAGCTACAACTTCCACAGCTTCAGCATCCGCATGGCCATCAGCCTGGGCCTGAACCGCGATCTGCCCAGCAGCTTCAGCGATAGCAGCATCCTGGAGCAGCGCCGCCGCATCTGGTGGAGCGTGTACAGCTGGGAGATCCAGCTGAGCCTGCTGTACGGCCGCAGCATCCAGCTGAGCCAGAACACCATCAGCTTCCCCAGCAGCGTGGATGATGTGCAGCGCACCACCACCGGCCCCACCATCTACCACGGCATCATCGAGACTGCCCGCCTGCTGCAGGTGTTCACCAAGATCTACGAGCTGGATAAGACCGTGACCGCCGAGAAGAGCCCCATCTGCGCCAAGAAGTGCCTGATGATCTGCAACGAGATCGAGGAGGTGAGCCGCCAGGCCCCCAAGTTCCTGCAGATGGATATCAGCACCACCGCCCTGACCAACCTGCTGAAGGAGCACCCCTGGCTGAGCTTCACCCGCTTCGAGCTGAAGTGGAAGCAGCTGAGCCTGATCATCTACGTGCTGCGCGATTTCTTCACCAACTTCACCCAGAAGAAGAGCCAGCTGGAGCAGGATCAGAACGATCACCAGAGCTACGAGGTGAAGCGCTGCAGCATCATGCTGAGCGATGCCGCCCAGCGCACCGTGATGAGCGTGAGCAGCTACATGGATAACCACAACGTGACCCCCTACTTCGCCTGGAACTGCAGCTACTACCTGTTCAACGCCGTGCTGGTGCCCATCAAGACCCTGCTGAGCAACAGCAAGAGCAACGCCGAGAACAACGAAACGGCCCAGCTGCTGCAGCAGATCAACACCGTGCTGATGCTGCTGAAGAAGCTGGCCACCTTCAAGATCCAGACCTGCGAGAAGTACATCCAGGTGCTGGAGGAGGTGTGCGCCCCCTTCCTGCTGAGCCAGTGCGCCATCCCCCTGCCCCACATCAGCTACAACAACAGCAACGGCAGCGCCATCAAGAACATCGTGGGCAGCGCCACCATCGCCCAGTACCCCACCCTGCCCGAGGAGAACGTGAACAACATCAGCGTGAAGTACGTGAGCCCCGGCAGCGTGGGACCCAGCCCCGTGCCCCTGAAGAGCGGCGCCAGCTTCAGCGATCTGGTGAAGCTGCTGAGCAACCGCCCCCCCAGCCGCAACAGCCCCGTGACCATCCCCCGCAGCACCCCCAGCCACCGCAGCGTGACCCCCTTCCTGGGCCAGCAGCAGCAGCTGCAGAGCCTGGTGCCCCTGACCCCCAGCGCCCTGTTCGGCGGCGCCAACTTCAACCAGAGCGGCAACATCGCCGATAGCAGCCTGAGCTTCACCTTCACCAACAGCAGCAACGGCCCCAACCTGATCACCACCCAGACCAACAGCCAGGCCCTGAGCCAGCCCATCGCCAGCAGCAACGTGCACGATAACTTCATGAACAACGAGATCACCGCCAGCAAGATCGATGATGGCAACAACAGCAAGCCCCTGAGCCCCGGCTGGACCGATCAGACCGCCTACAACGCCTTCGGCATCACCACCGGCATGTTCAACACCACCACCATGGATGATGTGTACAACTACCTGTTCGATGATGAGGATACCCCCCCCAACCCCAAGAAGGAGTAATAAGGCGCGCCCAAAGATCCAGACATGATAAGATACATTGATGAGTTTGGACAAACCACAACTAGAATGCAGTGAAAAAAATGCTTTATTTGTGAAATTTGTGATGCTATTGCTTTATTTGTAACCATTATAAGCTGCAATAAACAAGTTAACAACAACAATTGCATTCATTTTATGTTTCAGGTTCAGGGGGAGGTGTGGGAGGTTTTTTAAAGCAAGTAAAACCTCTACAAATGTGGTATGGCTGATTATGATCATAATTCGAATTCGAAGTTCCTATTCTCTAGAAAGTATAGGAACTTCGCCCGGGGATCTAATTCAATTAGAGACTAATTCAATTAGAGCTAATTCAATTAGGATCCAAGCTTATCGATTTCGAACCCTCGACCGCCGGAGTATAAATAGAGGCGCTTCGTCTACGGAGCGACAATTCAATTCAAACAAGCAAAGTGAACACGTCGCTAAGCGAAAGCTAAGCAAATAAACAAGCGCAGCTGAACAAGCTAAACAATCGGACTAGAGCCGGTCGCCGGCCGGCCACCATGGTGTCCAAGGGCGAGGAGCTGTTCACCGGCGTGGTGCCAATTCTGGTGGAGCTGGATGGCGACGTGAACGGCCACAAGTTCAGCGTGTCCGGCGAGGGCGAGGGCGACGCCACCTATGGAAAGCTGACCCTGAAGTTCATCTGCACCACCGGCAAGCTGCCCGTGCCATGGCCAACCCTCGTGACCACCCTGACCTATGGCGTGCAGTGCTTCAGCCGCTACCCCGATCACATGAAGCAGCACGATTTCTTCAAGAGCGCCATGCCCGAGGGCTACGTGCAGGAGCGCACCATCTTTTTCAAGGATGACGGCAACTACAAGACCCGCGCCGAAGTGAAGTTCGAGGGCGATACCCTCGTGAACCGCATCGAGCTGAAGGGCATCGATTTCAAGGAGGATGGAAACATCCTGGGCCACAAGCTGGAGTACAACTACAACAGCCACAACGTGTACATCATGGCCGACAAGCAGAAGAACGGCATCAAGGCCAACTTCAAGATCCGCCACAACATCGAGGATGGCGGCGTGCAGCTGGCCGATCACTACCAGCAGAACACCCCAATCGGCGACGGCCCAGTGCTGCTGCCCGATAACCATTACCTGAGCACCCAGAGCGCCCTGAGCAAGGATCCCAACGAGAAGCGCGACCACATGGTGCTGCTGGAGTTTGTGACCGCCGCCGGCATTACCCTGGGCATGGATGAGCTGTACAAGTAGGATCCAGACATGATAAGATACATTGATGAGTTTGGACAAACCACAACTAGAATGCAGTGAAAAAAATGCTTTATTTGTGAAATTTGTGATGCTATTGCTTTATTTGTAACCATTATAAGCTGCAATAAACAAGTTAACAACAACAATTGCATTCATTTTATGTTTCAGGTTCAGGGGGAGGTGTGGGAGGTTTTTTAAAGCAAGTAAAACCTCTACAAATGTGGTATGGCTGATTATGATCAGAAGTTCCTATTCTCTAGAAAGTATAGGAACTTCTCGCGCTCGCGCGACTGACGGTCGTAAGCACCCGCGTACGTGCGCGTTGTCTTCCGCGTGAGACCCGCGTGAGACGGATATCAAGCTTATCGATACCGTCGACCTCGAGGGGGGGCCCGGTACCCAGCTTTTGTTCCCTTTAGTGAGGGTTAATTGCGCGCTTGGCGTAATCATGGTCATAGCTGTTTCCTGTGTGAAATTGTTATCCGCTCACAATTCCACACAACATACGAGCCGGAAGCATAAAGTGTAAAGCCTGGGGTGCCTAATGAGTGAGCTAACTCACATTAATTGCGTTGCGCTCACTGCCCGCTTTCCAGTCGGGAAACCTGTCGTGCCAGCTGCATTAATGAATCGGCCAACGCGCGGGGAGAGGCGGTTTGCGTATTGGGCGCTCTTCCGCTTCCTCGCTCACTGACTCGCTGCGCTCGGTCGTTCGGCTGCGGCGAGCGGTATCAGCTCACTCAAAGGCGGTAATACGGTTATCCACAGAATCAGGGGATAACGCAGGAAAGAACATGTGAGCAAAAGGCCAGCAAAAGGCCAGGAACCGTAAAAAGGCCGCGTTGCTGGCGTTTTTCCATAGGCTCCGCCCCCCTGACGAGCATCACAAAAATCGACGCTCAAGTCAGAGGTGGCGAAACCCGACAGGACTATAAAGATACCAGGCGTTTCCCCCTGGAAGCTCCCTCGTGCGCTCTCCTGTTCCGACCCTGCCGCTTACCGGATACCTGTCCGCCTTTCTCCCTTCGGGAAGCGTGGCGCTTTCTCATAGCTCACGCTGTAGGTATCTCAGTTCGGTGTAGGTCGTTCGCTCCAAGCTGGGCTGTGTGCACGAACCCCCCGTTCAGCCCGACCGCTGCGCCTTATCCGGTAACTATCGTCTTGAGTCCAACCCGGTAAGACACGACTTATCGCCACTGGCAGCAGCCACTGGTAACAGGATTAGCAGAGCGAGGTATGTAGGCGGTGCTACAGAGTTCTTGAAGTGGTGGCCTAACTACGGCTACACTAGAAGGACAGTATTTGGTATCTGCGCTCTGCTGAAGCCAGTTACCTTCGGAAAAAGAGTTGGTAGCTCTTGATCCGGCAAACAAACCACCGCTGGTAGCGGTGGTTTTTTTGTTTGCAAGCAGCAGATTACGCGCAGAAAAAAAGGATCTCAAGAAGATCCTTTGATCTTTTCTACGGGGTCTGACGCTCAGTGGAACGAAAACTCACGTTAAGGGATTTTGGTCATGAGATTATCAAAAAGGATCTTCACCTAGATCCTTTTAAATTAAAAATGAAGTTTTAAATCAATCTAAAGTATATATGAGTAAACTTGGTCTGACAGTTACCAATGCTTAATCAGTGAGGCACCTATCTCAGCGATCTGTCTATTTCGTTCATCCATAGTTGCCTGACTCCCCGTCGTGTAGATAACTACGATACGGGAGGGCTTACCATCTGGCCCCAGTGCTGCAATGATACCGCGTGACCCACGCTCACCGGCTCCAGATTTATCAGCAATAAACCAGCCAGCCGGAAGGGCCGAGCGCAGAAGTGGTCCTGCAACTTTATCCGCCTCCATCCAGTCTATTAATTGTTGCCGGGAAGCTAGAGTAAGTAGTTCGCCAGTTAATAGTTTGCGCAACGTTGTTGCCATTGCTACAGGCATCGTGGTGTCACGCTCGTCGTTTGGTATGGCTTCATTCAGCTCCGGTTCCCAACGATCAAGGCGAGTTACATGATCCCCCATGTTGTGCAAAAAAGCGGTTAGCTCCTTCGGTCCTCCGATCGTTGTCAGAAGTAAGTTGGCCGCAGTGTTATCACTCATGGTTATGGCAGCACTGCATAATTCTCTTACTGTCATGCCATCCGTAAGATGCTTTTCTGTGACTGGTGAGTACTCAACCAAGTCATTCTGAGAATAGTGTATGCGGCGACCGAGTTGCTCTTGCCCGGCGTCAATACGGGATAATACCGCGCCACATAGCAGAACTTTAAAAGTGCTCATCATTGGAAAACGTTCTTCGGGGCGAAAACTCTCAAGGATCTTACCGCTGTTGAGATCCAGTTCGATGTAACCCACTCGTGCACCCAACTGATCTTCAGCATCTTTTACTTTCACCAGCGTTTCTGGGTGAGCAAAAACAGGAAGGCAAAATGCCGCAAAAAAGGGAATAAGGGCGACACGGAAATGTTGAATACTCATACTCTTCCTTTTTCAATATTATTGAAGCATTTATCAGGGTTATTGTCTCATGAGCGGATACATATTTGAATGTATTTAGAAAAATAAACAAATAGGGGTTCCGCGCACATTTCCCCGAAAAGTGCCAC

FRT SV40polyA GFP 3XP3-Hs70 promoter M13Frw primer KozakGAL4 AmpR

pM37_SA_KozakGAL4

CTAAATTGTAAGCGTTAATATTTTGTTAAAATTCGCGTTAAATTTTTGTTAAATCAGCTCATTTTTTAACCAATAGGCCGAAATCGGCAAAATCCCTTATAAATCAAAAGAATAGACCGAGATAGGGTTGAGTGTTGTTCCAGTTTGGAACAAGAGTCCACTATTAAAGAACGTGGACTCCAACGTCAAAGGGCGAAAAACCGTCTATCAGGGCGATGGCCCACTACGTGAACCATCACCCTAATCAAGTTTTTTGGGGTCGAGGTGCCGTAAAGCACTAAATCGGAACCCTAAAGGGAGCCCCCGATTTAGAGCTTGACGGGGAAAGCCGGCGAACGTGGCGAGAAAGGAAGGGAAGAAAGCGAAAGGAGCGGGCGCTAGGGCGCTGGCAAGTGTAGCGGTCACGCTGCGCGTAACCACCACACCCGCCGCGCTTAATGCGCCGCTACAGGGCGCGTCCCATTCGCCATTCAGGCTGCGCAACTGTTGGGAAGGGCGATCGGTGCGGGCCTCTTCGCTATTACGCCAGCTGGCGAAAGGGGGATGTGCTGCAAGGCGATTAAGTTGGGTAACGCCAGGGTTTTCCCAGTCACGACGTTGTAAAACGACGGCCAGTGAGCGCGCGTAATACGACTCACTATAGGGCGAATTGGAGCTCCACCGCGGTGGCGTCTCTAAACGGTCTCTAAACGAAGACTTAAACAGTCGATCCAACATGGCGACTTGTCCCATCCCCGGCATGTTTAAATATACTAATTATTCTTGAACTAATTTTAATCAACCGATTTATCTCTCTTCCGCAGCCAAAATGAAGCTGCTGAGCAGCATCGAGCAGGCCTGCGATATCTGCCGCCTGAAGAAGCTGAAGTGCAGCAAGGAGAAGCCCAAGTGCGCCAAGTGCCTGAAGAACAACTGGGAGTGCCGCTACAGCCCCAAGACCAAGCGCAGCCCCCTGACCCGCGCCCACCTGACCGAGGTGGAGAGCCGCCTGGAGCGCCTGGAGCAGCTGTTCCTGCTGATCTTCCCCCGCGAGGATCTGGATATGATCCTGAAGATGGATAGCCTGCAGGATATCAAGGCCCTGCTGACCGGCCTGTTCGTGCAGGATAACGTGAACAAGGATGCCGTGACCGATCGCCTGGCCAGCGTGGAAACCGATATGCCCCTGACCCTGCGCCAGCACCGCATCAGCGCCACCAGCAGCAGCGAGGAGAGCAGCAACAAGGGCCAGCGCCAGCTGACCGTGAGCATCGATAGCGCCGCCCACCACGATAACAGCACCATCCCCCTGGATTTCATGCCCCGCGATGCCCTGCACGGCTTCGATTGGAGCGAGGAGGATGATATGAGCGATGGCCTGCCCTTCCTGAAAACCGATCCCAACAACAACGGCTTCTTCGGCGATGGCAGCCTGCTGTGCATCCTGCGCAGCATCGGCTTCAAGCCCGAGAACTACACCAACAGCAACGTGAACCGCCTGCCCACCATGATCACCGATCGCTACACCCTGGCCAGCCGCAGCACCACCAGCCGCCTGCTGCAGAGCTACCTGAACAACTTCCACCCCTACTGCCCCATCGTGCACAGCCCCACCCTGATGATGCTGTACAACAACCAGATCGAGATCGCCAGCAAGGATCAGTGGCAGATCCTGTTCAACTGCATCCTGGCCATCGGCGCCTGGTGCATCGAGGGCGAGAGCACCGATATCGATGTGTTCTACTACCAGAACGCCAAGAGCCACCTGACCAGCAAGGTGTTCGAGAGCGGCAGCATCATCCTGGTGACCGCCCTGCACCTGCTGAGCCGCTACACCCAGTGGCGCCAAAAGACCAACACCAGCTACAACTTCCACAGCTTCAGCATCCGCATGGCCATCAGCCTGGGCCTGAACCGCGATCTGCCCAGCAGCTTCAGCGATAGCAGCATCCTGGAGCAGCGCCGCCGCATCTGGTGGAGCGTGTACAGCTGGGAGATCCAGCTGAGCCTGCTGTACGGCCGCAGCATCCAGCTGAGCCAGAACACCATCAGCTTCCCCAGCAGCGTGGATGATGTGCAGCGCACCACCACCGGCCCCACCATCTACCACGGCATCATCGAGACTGCCCGCCTGCTGCAGGTGTTCACCAAGATCTACGAGCTGGATAAGACCGTGACCGCCGAGAAGAGCCCCATCTGCGCCAAGAAGTGCCTGATGATCTGCAACGAGATCGAGGAGGTGAGCCGCCAGGCCCCCAAGTTCCTGCAGATGGATATCAGCACCACCGCCCTGACCAACCTGCTGAAGGAGCACCCCTGGCTGAGCTTCACCCGCTTCGAGCTGAAGTGGAAGCAGCTGAGCCTGATCATCTACGTGCTGCGCGATTTCTTCACCAACTTCACCCAGAAGAAGAGCCAGCTGGAGCAGGATCAGAACGATCACCAGAGCTACGAGGTGAAGCGCTGCAGCATCATGCTGAGCGATGCCGCCCAGCGCACCGTGATGAGCGTGAGCAGCTACATGGATAACCACAACGTGACCCCCTACTTCGCCTGGAACTGCAGCTACTACCTGTTCAACGCCGTGCTGGTGCCCATCAAGACCCTGCTGAGCAACAGCAAGAGCAACGCCGAGAACAACGAAACGGCCCAGCTGCTGCAGCAGATCAACACCGTGCTGATGCTGCTGAAGAAGCTGGCCACCTTCAAGATCCAGACCTGCGAGAAGTACATCCAGGTGCTGGAGGAGGTGTGCGCCCCCTTCCTGCTGAGCCAGTGCGCCATCCCCCTGCCCCACATCAGCTACAACAACAGCAACGGCAGCGCCATCAAGAACATCGTGGGCAGCGCCACCATCGCCCAGTACCCCACCCTGCCCGAGGAGAACGTGAACAACATCAGCGTGAAGTACGTGAGCCCCGGCAGCGTGGGACCCAGCCCCGTGCCCCTGAAGAGCGGCGCCAGCTTCAGCGATCTGGTGAAGCTGCTGAGCAACCGCCCCCCCAGCCGCAACAGCCCCGTGACCATCCCCCGCAGCACCCCCAGCCACCGCAGCGTGACCCCCTTCCTGGGCCAGCAGCAGCAGCTGCAGAGCCTGGTGCCCCTGACCCCCAGCGCCCTGTTCGGCGGCGCCAACTTCAACCAGAGCGGCAACATCGCCGATAGCAGCCTGAGCTTCACCTTCACCAACAGCAGCAACGGCCCCAACCTGATCACCACCCAGACCAACAGCCAGGCCCTGAGCCAGCCCATCGCCAGCAGCAACGTGCACGATAACTTCATGAACAACGAGATCACCGCCAGCAAGATCGATGATGGCAACAACAGCAAGCCCCTGAGCCCCGGCTGGACCGATCAGACCGCCTACAACGCCTTCGGCATCACCACCGGCATGTTCAACACCACCACCATGGATGATGTGTACAACTACCTGTTCGATGATGAGGATACCCCCCCCAACCCCAAGAAGGAGTAATAAGGCGCGCCCAAAGATCCAGACATGATAAGATACATTGATGAGTTTGGACAAACCACAACTAGAATGCAGTGAAAAAAATGCTTTATTTGTGAAATTTGTGATGCTATTGCTTTATTTGTAACCATTATAAGCTGCAATAAACAAGTTAACAACAACAATTGCATTCATTTTATGTTTCAGGTTCAGGGGGAGGTGTGGGAGGTTTTTTAAAGCAAGTAAAACCTCTACAAATGTGGTATGGCTGATTATGATCATAATTCGAATTCGAAGTTCCTATTCTCTAGAAAGTATAGGAACTTCGCCCGGGGATCTAATTCAATTAGAGACTAATTCAATTAGAGCTAATTCAATTAGGATCCAAGCTTATCGATTTCGAACCCTCGACCGCCGGAGTATAAATAGAGGCGCTTCGTCTACGGAGCGACAATTCAATTCAAACAAGCAAAGTGAACACGTCGCTAAGCGAAAGCTAAGCAAATAAACAAGCGCAGCTGAACAAGCTAAACAATCGGACTAGAGCCGGTCGCCGGCCGGCCACCATGGTGTCCAAGGGCGAGGAGCTGTTCACCGGCGTGGTGCCAATTCTGGTGGAGCTGGATGGCGACGTGAACGGCCACAAGTTCAGCGTGTCCGGCGAGGGCGAGGGCGACGCCACCTATGGAAAGCTGACCCTGAAGTTCATCTGCACCACCGGCAAGCTGCCCGTGCCATGGCCAACCCTCGTGACCACCCTGACCTATGGCGTGCAGTGCTTCAGCCGCTACCCCGATCACATGAAGCAGCACGATTTCTTCAAGAGCGCCATGCCCGAGGGCTACGTGCAGGAGCGCACCATCTTTTTCAAGGATGACGGCAACTACAAGACCCGCGCCGAAGTGAAGTTCGAGGGCGATACCCTCGTGAACCGCATCGAGCTGAAGGGCATCGATTTCAAGGAGGATGGAAACATCCTGGGCCACAAGCTGGAGTACAACTACAACAGCCACAACGTGTACATCATGGCCGACAAGCAGAAGAACGGCATCAAGGCCAACTTCAAGATCCGCCACAACATCGAGGATGGCGGCGTGCAGCTGGCCGATCACTACCAGCAGAACACCCCAATCGGCGACGGCCCAGTGCTGCTGCCCGATAACCATTACCTGAGCACCCAGAGCGCCCTGAGCAAGGATCCCAACGAGAAGCGCGACCACATGGTGCTGCTGGAGTTTGTGACCGCCGCCGGCATTACCCTGGGCATGGATGAGCTGTACAAGTAGGATCCAGACATGATAAGATACATTGATGAGTTTGGACAAACCACAACTAGAATGCAGTGAAAAAAATGCTTTATTTGTGAAATTTGTGATGCTATTGCTTTATTTGTAACCATTATAAGCTGCAATAAACAAGTTAACAACAACAATTGCATTCATTTTATGTTTCAGGTTCAGGGGGAGGTGTGGGAGGTTTTTTAAAGCAAGTAAAACCTCTACAAATGTGGTATGGCTGATTATGATCAGAAGTTCCTATTCTCTAGAAAGTATAGGAACTTCTCGCGCTCGCGCGACTGACGGTCGTAAGCACCCGCGTACGTGCGCGTTGTCTTCCGCGTGAGACCCGCGTGAGACGGATATCAAGCTTATCGATACCGTCGACCTCGAGGGGGGGCCCGGTACCCAGCTTTTGTTCCCTTTAGTGAGGGTTAATTGCGCGCTTGGCGTAATCATGGTCATAGCTGTTTCCTGTGTGAAATTGTTATCCGCTCACAATTCCACACAACATACGAGCCGGAAGCATAAAGTGTAAAGCCTGGGGTGCCTAATGAGTGAGCTAACTCACATTAATTGCGTTGCGCTCACTGCCCGCTTTCCAGTCGGGAAACCTGTCGTGCCAGCTGCATTAATGAATCGGCCAACGCGCGGGGAGAGGCGGTTTGCGTATTGGGCGCTCTTCCGCTTCCTCGCTCACTGACTCGCTGCGCTCGGTCGTTCGGCTGCGGCGAGCGGTATCAGCTCACTCAAAGGCGGTAATACGGTTATCCACAGAATCAGGGGATAACGCAGGAAAGAACATGTGAGCAAAAGGCCAGCAAAAGGCCAGGAACCGTAAAAAGGCCGCGTTGCTGGCGTTTTTCCATAGGCTCCGCCCCCCTGACGAGCATCACAAAAATCGACGCTCAAGTCAGAGGTGGCGAAACCCGACAGGACTATAAAGATACCAGGCGTTTCCCCCTGGAAGCTCCCTCGTGCGCTCTCCTGTTCCGACCCTGCCGCTTACCGGATACCTGTCCGCCTTTCTCCCTTCGGGAAGCGTGGCGCTTTCTCATAGCTCACGCTGTAGGTATCTCAGTTCGGTGTAGGTCGTTCGCTCCAAGCTGGGCTGTGTGCACGAACCCCCCGTTCAGCCCGACCGCTGCGCCTTATCCGGTAACTATCGTCTTGAGTCCAACCCGGTAAGACACGACTTATCGCCACTGGCAGCAGCCACTGGTAACAGGATTAGCAGAGCGAGGTATGTAGGCGGTGCTACAGAGTTCTTGAAGTGGTGGCCTAACTACGGCTACACTAGAAGGACAGTATTTGGTATCTGCGCTCTGCTGAAGCCAGTTACCTTCGGAAAAAGAGTTGGTAGCTCTTGATCCGGCAAACAAACCACCGCTGGTAGCGGTGGTTTTTTTGTTTGCAAGCAGCAGATTACGCGCAGAAAAAAAGGATCTCAAGAAGATCCTTTGATCTTTTCTACGGGGTCTGACGCTCAGTGGAACGAAAACTCACGTTAAGGGATTTTGGTCATGAGATTATCAAAAAGGATCTTCACCTAGATCCTTTTAAATTAAAAATGAAGTTTTAAATCAATCTAAAGTATATATGAGTAAACTTGGTCTGACAGTTACCAATGCTTAATCAGTGAGGCACCTATCTCAGCGATCTGTCTATTTCGTTCATCCATAGTTGCCTGACTCCCCGTCGTGTAGATAACTACGATACGGGAGGGCTTACCATCTGGCCCCAGTGCTGCAATGATACCGCGTGACCCACGCTCACCGGCTCCAGATTTATCAGCAATAAACCAGCCAGCCGGAAGGGCCGAGCGCAGAAGTGGTCCTGCAACTTTATCCGCCTCCATCCAGTCTATTAATTGTTGCCGGGAAGCTAGAGTAAGTAGTTCGCCAGTTAATAGTTTGCGCAACGTTGTTGCCATTGCTACAGGCATCGTGGTGTCACGCTCGTCGTTTGGTATGGCTTCATTCAGCTCCGGTTCCCAACGATCAAGGCGAGTTACATGATCCCCCATGTTGTGCAAAAAAGCGGTTAGCTCCTTCGGTCCTCCGATCGTTGTCAGAAGTAAGTTGGCCGCAGTGTTATCACTCATGGTTATGGCAGCACTGCATAATTCTCTTACTGTCATGCCATCCGTAAGATGCTTTTCTGTGACTGGTGAGTACTCAACCAAGTCATTCTGAGAATAGTGTATGCGGCGACCGAGTTGCTCTTGCCCGGCGTCAATACGGGATAATACCGCGCCACATAGCAGAACTTTAAAAGTGCTCATCATTGGAAAACGTTCTTCGGGGCGAAAACTCTCAAGGATCTTACCGCTGTTGAGATCCAGTTCGATGTAACCCACTCGTGCACCCAACTGATCTTCAGCATCTTTTACTTTCACCAGCGTTTCTGGGTGAGCAAAAACAGGAAGGCAAAATGCCGCAAAAAAGGGAATAAGGGCGACACGGAAATGTTGAATACTCATACTCTTCCTTTTTCAATATTATTGAAGCATTTATCAGGGTTATTGTCTCATGAGCGGATACATATTTGAATGTATTTAGAAAAATAAACAAATAGGGGTTCCGCGCACATTTCCCCGAAAAGTGCCAC

FRT SV40polyA GFP 3XP3-Hs70 promoter M13Frw primer Splice Acceptor KozakGAL4 AmpR
